# Meescan

**DOI:** 10.29173/jchla29605

**Published:** 2022-04-01

**Authors:** Victoria Eke

**Affiliations:** Scholarly Communications Librarian Concordia University of Edmonton Edmonton, Alberta, Canada

**Product:** Meescan

**URL:** https://www.meescan.com

## Purpose and Intended Audience

Based in Maple Ridge, British Columbia, Meescan offers flexible, user-friendly self-checkout solutions for various institution types, including academic, hospital, and special libraries. Partner libraries benefit from continuous and personalized customer service support, and patrons can easily navigate the self-checkout software at on-site kiosks or through the mobile app. With many customizable options, from kiosk design to system configuration, Meescan can be adapted to fit the specific needs of libraries of all kinds and sizes.

## Product Description

Promising to “liberate librarians away from the checkout desk so they can do what they do best,” Meescan offers self-checkout kiosk and security stations that require little to no hardware and minimal floor space.

Meescan kiosks are available in three types: floor, counter, and wall mounted. The floor type includes a stand on which the iPad enclosure is attached. The counter allows more space for the patron to rest checkout items and personal belongings. The wall mounted requires no floor space and, in libraries where multiple kiosks are available, can be installed at different heights.

All kiosks are equipped with a CCD (charge coupled device) barcode scanner for scanning patron ID cards and items. With numerous symbologies supported, the built-in scanner is configured during the implementation phase depending on the needs of the library. Additional kiosk reader types are available for libraries that use magnetic stripe or contactless cards to authenticate patrons [1].

The free Meescan app, available to download from the App Store and Google Play Store, offers a mobile self-checkout option that allows patrons to check items out privately from anywhere in the library.

Designed as a cloud-based service that is managed fully via online network, Meescan provides reliable, secure, and adaptable service requiring no on-site installation or repairs.

## Special Features

- Mobile self-checkout: The downloadable app for Apple and Android devices, which uses Bluetooth and geolocation technology allows patrons to check items out privately from anywhere in the library [2].
- Custom branding for the mobile app, kiosks, and marketing materials is available for an additional annual cost.
- Designed as a cloud-based service that is managed fully via online network, Meescan’s distributed data centre and servers provide reliable, secure and compliant service to libraries all over the world, requiring no on-site installation or repairs.

## Compatibility

Meescan kiosks require an Apple iPad, which is supplied by the library. Currently, the 10.2-inch iPad (9th generation) is recommended, but kiosks are compatible with most iPad models from 2018 and newer, including iPad Air, iPad mini, and iPad Pro [3].

Meescan is available as a mobile app for iPhone (requires iOS 9.0 or later), iPad (requires iOS 9.0 or later), and Android (4.1 and up) devices.

Meescan is compatible with electro-magnetic (EM), radio-frequency identification (RFID), and passaround security. Security stations can be used by patrons using a kiosk or the app [3].

For patron authentication, Meescan supports a variety of barcode symbologies (Codabar, Code 39, Code 11, Code 93, Code 128, Interleaved 2 of 5, MSI, and Databar), contactless cards (near-field communication (NFC), tap, etc.), single sign-on, magnetic stripe, and manual entry [1].

## Implementation

The implementation phase averages 4-6 weeks from the time of ordering to the arrival of equipment. Throughout the process, the Meescan provides a dedicated onboarding team with all the information necessary to configure Meescan with the library’s ILS through a secure SIP2 connection, such as library locations, item barcode types and barcode positioning on items, patron credentials, and connectivity specifications. Once the kiosk is received by the library, the Meescan app is downloaded, the iPad is configured for public use and is placed inside the kiosk enclosure. This ‘plug and play’ method of installation eliminates the need for costly set-up by an on-site technician. The Meescan support team is ready to assist by phone, email, or chat throughout the installation and testing phases of implementation. Following the purchase of equipment, Meescan offers flexible pricing based on patronage.

## Usability

**Fig. 1:**
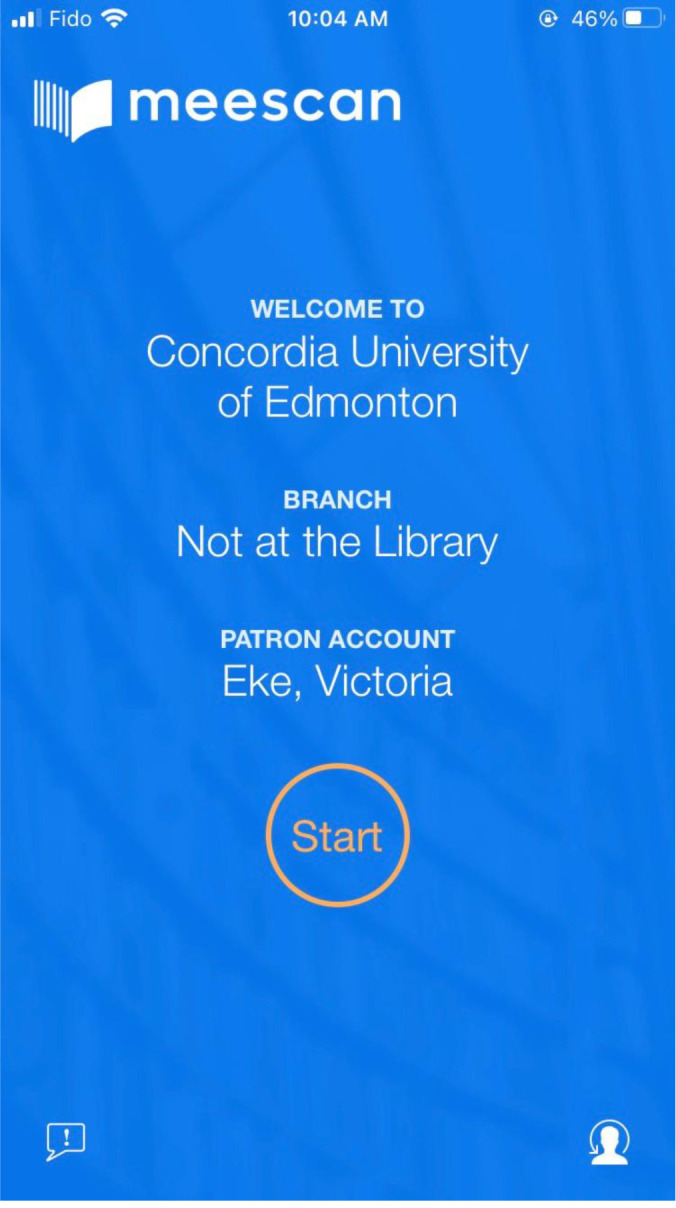
A screenshot of the Meescan app welcome screen.

The Meescan app (Figure 1) can be downloaded to iOS and Android devices free of charge. On personal devices, patrons are required to authenticate only the first time they use the Meescan app. Subsequently, the app will remember them, eliminating the need for library cards or student IDs for each transaction [1].

The app’s simple yet stylish design and guided prompts provide the patron with a user-friendly interface and streamlined experience from beginning to end. While checkout service is available only when the patron is in the library with location services enabled, account information, including number of current loans and fees due, is available at any time.

On-site kiosks are just as easy to use, providing patrons with the same guided checkout experience they would receive using the mobile app.

## Strengths and Weaknesses

### Strengths

- An annual Meescan license includes the use of the app on both patrons’ personal devices and on the Meescan kiosk(s) in your library.
- Maintenance, technical support, and warranty are included with an annual license at no additional cost.
- Meescan does not retain any personal information pertaining to databases or patrons - all identifying information is encrypted.
- Meescan’s online chat service allows current and prospective users to immediately connect with a member of the team for general information and inquiries.

### Weaknesses

- Issues/errors with iOS updates can temporarily affect the functionality of the kiosk.

## Comparison with Similar Products

Minnesota-based [4] Biblioteca provides a multitude of library solutions to institutions worldwide, including self-checkout options [5] not unlike those offered by Meescan. Overall, Biblioteca’s self-checkout offerings, such as a mobile app and numerous kiosk models and configurations from which to choose, are comparable to the solutions offered by Meescan. British Columbia-based Meescan, however, offers a more focused and personalized service with designated and continuous support that is largely unmatched in larger companies with a broader scope for services rendered.

## Cost

- License**:** For academic and special libraries, annual license costs, based on full-time enrolment (FTE) or the number of patrons served by each library location, start at $699. For institutions with numerous library locations or branches, site-wide license options allow for the use of Meescan in any and all locations.
- Equipment: The different kiosk types, floor-, counter-, and wall-mounted, range from $599 to $829, and the library would provide an iPad to place inside the kiosk. Security stations for desensitizing items with EM strips or RFID tags are $2,395 each. All equipment, including kiosks and security stations, are one-time purchases

## Conclusion

Overall, Meescan offers flexible, user-friendly self-checkout solutions that can be utilized in academic, hospital, and other specialized health sciences libraries of any size. With many options for customization and ongoing and personalized support from the Meescan team, partner libraries can feel confident in their choice to implement a system adaptable to the specific needs of the institution. Moreover, patrons can enjoy a streamlined process for checking out items privately from anywhere in the library through the mobile app available on a number of devices and platforms. With continued innovation and product development, exemplary customer service and support, and flexible pricing options, Meescan is a top option for Canadian health sciences libraries.
